# Foster Care Involvement Among Youth With Intellectual and Developmental Disabilities

**DOI:** 10.1001/jamapediatrics.2023.6580

**Published:** 2024-02-12

**Authors:** Lindsay Shea, Melissa L. Villodas, Jonas Ventimiglia, Amy Blank Wilson, Dylan Cooper

**Affiliations:** 1 Policy and Analytics Center, A.J. Drexel Autism Institute, Philadelphia, Pennsylvania; 2 Department of Social Work, George Mason University, Fairfax, Virginia; 3 School of Social Work, University of North Carolina at Chapel Hill, Chapel Hill

## Abstract

**Importance:**

Youth with intellectual and developmental disabilities (I/DD) are more likely to be placed in foster care than other youth. Examining the clinical and sociodemographic characteristics of youth with I/DD in the foster care system is critical for identifying disparities and understanding service needs.

**Objective:**

To produce a population-level analysis of youth with I/DD in foster care that examines differences in rates of foster care involvement based on race, ethnicity, age, and sex.

**Design, Setting, and Participants:**

This cross-sectional study involved all individuals with I/DD 21 years and younger enrolled in Medicaid through foster care in 2016 via data from Transformed Medicaid Statistical Information System (T-MSIS) Analytic Files (TAF) for all 50 US states and Washington, DC. As a key insurer of I/DD services and foster care, Medicaid claims offer a timely population-level analysis. Youth with I/DD were grouped into diagnostic subgroups: autism spectrum disorder (ASD) only, intellectual disability only, or ASD and ID. The data analysis took place from July 2022 to September 2023.

**Exposure:**

TAF data contain Medicaid enrollment information by month with a binary indicator of foster care involvement, and eligibility files identify race, ethnicity, age, and sex.

**Main Outcomes and Measures:**

The period prevalence of foster care involvement was determined among I/DD youth by diagnostic subgroups using an intersectional approach across race, ethnicity, age, and sex. Logistic regression examined associations between risk for foster care involvement and race, ethnicity, age, and sex.

**Results:**

A total of 39 143 youth with I/DD had foster care involvement in 2016. Black youth (adjusted odds ratio [aOR], 1.37; 95% CI, 1.28-1.47) and females (aOR, 1.18; 95% CI, 1.1-1.27) had increased likelihood for foster care involvement. The likelihood for foster care involvement increased with age in all groups relative to the age group 0 to 5 years old.

**Conclusions and Relevance:**

This study found that among youth with I/DD, Black youth and females faced higher risk for foster care involvement, and the likelihood of foster care involvement increased with age. There is an urgent need for research that focuses on addressing system-level factors that drive increased risk. Understanding the specific health needs of Black and female youth with I/DD is critical to ensure the formation, implementation, and monitoring of equitable delivery of health services.

## Key Points

**Question:**

Is foster care involvement among youth with intellectual and developmental disabilities (I/DD) increasing and to what extent are subgroups disproportionately affected?

**Findings:**

This cross-sectional study found that the population of youth with I/DD in foster care has grown compared with previous research. Black youth and females faced higher risk for foster care involvement, compared with youth who were White or male, and the likelihood of foster care involvement increased with age.

**Meaning:**

Findings suggest that foster care involvement for youth with I/DD differs based on race and sex, which has important implications for equitable health outcomes as the number of youth with I/DD and foster care involvement increases.

## Teaser

This cross-sectional study examines the clinical and sociodemographic characteristics of individuals with intellectual and developmental disabilities 21 years and younger enrolled in Medicaid through foster care to assess differences in rates of foster care involvement based on race, ethnicity, age, and sex.

## Introduction

The American Academy of Pediatrics declared that children in foster care have special health care needs driven by complex and serious medical, mental health, and developmental issues associated with childhood adversity and trauma.^1^ Children enter foster care for many reasons, including abuse and neglect, non–maltreatment-related issues (eg, parental death or incarceration), and barriers related to accessing needed services and resources, including supports for caring for children with developmental or health care needs.^2,3^ Children enter foster care with an array of needs, including acute illnesses such as infections and injuries that are a direct result of abuse and neglect and chronic health conditions such as asthma and anemia, which are often undiagnosed or undertreated,^4^ in addition to developmental and intellectual disability diagnoses.^5^

Children diagnosed with autism spectrum disorder (ASD) or children with an intellectual disability (ID) diagnosis, collectively referred to as intellectual and developmental disabilities (I/DD), likely present with a range of service and support needs given the complexities of their diagnoses and numerous, co-occurring conditions.^6,7^ For children with I/DD, foster care involvement may pose additive risk for complex health care needs, given that delays or disruptions in accessing services can adversely affect overall health, developmental, and life trajectories.^8,9,10^ The foster care system may further exacerbate service barriers as multiple placements can lead to disruptions in services through changes in clinicians. Elevated odds for foster care involvement among youth with I/DD have been documented.^11^ Existing research suggests this may be the result of increased risk of neglect and abuse.^11,12^ Collectively, these factors catalyze a call for research on the prevalence and patterns of children with I/DD in foster care to identify opportunities to mitigate heightened risk for adverse health outcomes.

Most children with I/DD (those with and without foster care involvement) rely on Medicaid-funded services and supports, as it is the largest insurer of behavioral health services in the US.^13^ Dually, Medicaid permits children placed in foster care to automatically enroll. Preliminary research among Medicaid-enrolled children from 2001 to 2007 found that foster care entry was 2.4 times greater for youth with ASD than that of typically developing youth, 1.9 times greater for youth with ID, and 1.6 times greater for youth with ASD and co-occurring ID when compared with typically developing children.^11^ Since this time period, autism prevalence in the US has substantially increased. In 2004, 1 in 125 children aged 8 years met diagnostic criteria for ASD, while in 2020, 1 in 36 children aged 8 years had ASD.^14^ Recent estimates also suggest that 37.9% of children with ASD have a co-occurring ID,^15,16^ further emphasizing the complex health care needs of this population. Children diagnosed with ID but not ASD constitute roughly 1.2% of the US population.^17^

The increase in ASD prevalence can be explained in part by improved identification of ASD in historically marginalized populations. For instance, while White children have historically received ASD diagnoses earlier and at higher rates than Black and Latinx children,^18^ emerging reports suggest that trends in the identification of ASD among children from underserved backgrounds has increased in recent years, with Asian or Pacific Islander, Black, and Hispanic children recently having higher documented rates of identification than White children at 4 and 8 years old.^19^ This research also finds that Black children in particular continue to be disproportionately represented among children identified with ASD and ID.^20^ Research on racial and ethnic differences among children in foster care consistently finds that Black children and Native American and Alaskan Native children are overrepresented compared with the general population of children in the US.^21^ Differences by sex are dually prominent in the examination of I/DD and youth in foster care. While the rates of foster care involvement among males and females are similar to those found in the general population,^22^ previous estimates had suggested that ASD occurs at a 4:1 ratio between males and females.^23^ Recent research has shown that the core features of ASD may present differently for females,^24^ but how this diagnostic disparity impacts the foster care population remains unclear.

The goal of this study is to use a population-level, intersectional examination of foster care involvement for youth with I/DD to (1) quantify the number of youth with I/DD, by autism and ID subgroups, in the foster care system in 2016, and (2) identify differences in foster care involvement based on race, ethnicity, age, and sex.

## Methods

The Drexel University institutional review board approved a waiver for the use of claims data for this study. This study followed the Strengthening the Reporting of Observational Studies in Epidemiology (STROBE) reporting guideline.

National Medicaid claims data from all states and Washington, DC, were collected via the Transformed Medicaid Statistical Information System (T-MSIS) Analytic Files (TAF) in calendar year 2016. Medicaid claims data include indicators for individual demographic and Medicaid enrollment status by month, as well as service files with diagnosis and procedure codes and prescription medications. The sample included individuals enrolled for a minimum of 9 months to account for administrative churning. Additionally, individuals without a code indicating their reason for Medicaid enrollment were excluded (n = 45 386), and individuals with missing sex data were excluded because of the small sample size (<11). All youth 21 years and younger with ASD or ID diagnoses (or both) enrolled in Medicaid were placed into 3 subgroups: youth with an ASD diagnosis and no ID diagnosis (ASD only), youth with an ID diagnosis and no ASD diagnosis (ID only), and youth with both an ASD and an ID diagnosis (ASD + ID). Validated algorithms for identifying ASD and ID in claims data were applied from the Chronic Condition Warehouse.^25^ These algorithms require 1 inpatient claim or 2 other claims to account for coding practices and potential rule-out diagnosis especially in outpatient settings.

### Variables

TAF data include information about Medicaid eligibility that contains specific categories for youth who are eligible because they are enrolled in foster care, Title IV-E adoption assistance, or guardianship care. Race and ethnicity variables are captured within the demographic information (32% of the sample were missing these data so they were excluded). Age was calculated as of December 1, 2016, and grouped into 4 categories: pre–formal school (aged 0-5 years), early ages (6-12 years), teenage/postpuberty (13-17 years), and transition-age adults (18-21 years). Sex in the TAF data was described as male or female.

### Analysis

We examined the distribution of categorical demographic variables for the ASD-only, ID-only, and ASD + ID groups enrolled in foster care. We determined the prevalence of foster care involvement for each diagnosis group by race, ethnicity, age group, and sex across groups. We also established the prevalence of foster care involvement by intersectional categorizations across sex and race and ethnicity.^26^ Logistic regressions examined associations between the likelihood of foster care involvement and race, ethnicity, age, and sex adjusting for age separate from sex and race and ethnicity. Data analysis was conducted with R version 4.3 from July 2022 to September 2023.

## Results

A total of 39 143 youth with I/DD had foster care involvement in 2016. The ASD group (n = 28 442) was larger than the ID group (n = 16 125) by almost 2-fold. One in 5 youth with ASD and foster care involvement also had co-occurring ID (Table 1). Overall, among all youth with I/DD enrolled in Medicaid, 23 018 youth with ASD only (4.3%) had foster care involvement, compared with 10 701 youth with ASD and ID (4.6%) and 5424 youth with ID (5.1%).

**Table 1.** Sample Characteristics by Diagnosis Group

Characteristic	No. (%)		
	ASD only (n = 23 018)	ID only (n = 10 701)	ASD and ID (n = 5424)
Age group, y			
0-5	3061 (13.3)	449 (4.2)	103 (1.9)
6-12	10 404 (45.2)	3692 (34.5)	1784 (32.9)
13-17	7504 (32.6)	4377 (40.9)	2457 (45.3)
18-21	2049 (8.9)	2183 (20.4)	1079 (19.9)
Sex			
Male	16 251 (70.6)	5982 (55.9)	3884 (71.6)
Female	6767 (29.4)	4719 (44.1)	1540 (28.4)
Race and ethnicity			
American Indian and Alaska Native, non-Hispanic^a^	NA	NA	NA
Asian or Pacific Islander^a^	115 (0.5)	NA	33 (0.6)
Black or African American	4005 (17.4)	3103 (29.0)	1269 (23.4)
Hispanic or Latino	230 (1.0)	128 (1.2)	65 (1.2)
White	9967 (43.3)	4462 (41.7)	2609 (48.1)
More than 1 race or ethnicity^a^	NA	214 (2.0)	NA
Missing	8632 (37.5)	2665 (24.9)	1416 (26.1)

Abbreviations: ASD, autism spectrum disorder; ID, intellectual disability; NA, not available.

^a^ Values censored for compliance with cell suppression policy; all are less than 0.2%.

In alignment with national ASD prevalence estimates, foster care–involved youth with an ASD diagnosis were more likely to be male (Table 2). Across all groups, being female was associated with increased risk for foster care involvement, with the highest risk presenting among the ASD-only group (adjusted odds ratio [aOR], 1.35; 95% CI, 1.39-1.4). In terms of race and ethnicity, all diagnosis groups were predominantly White compared with the other racial and ethnic groups. However, a sizable proportion of all groups was Black, totaling more than 6400 (27.9%) foster care–involved youth with ASD, 1735 (32%) foster care–involved youth with ASD and ID, and 4173 (39%) foster care–involved youth with ID only. Black youth in all groups were also at increased risk for foster care involvement (aOR, 1.37; 95% CI, 1.32-1.42, for those with ASD only; aOR, 1.37; 95% CI, 1.28-1.47, for ASD and ID; and aOR, 2.32; 95% CI, 1.26-1.39, for ID only).

**Table 2.** Intersection of Race and Ethnicity and Sex by Diagnosis Group^a^

Characteristic	ASD only (n = 23 018), %	ID only (n = 10 701), %	ASD and ID (n = 5424), %
Male			
American Indian and Alaska Native, non-Hispanic	0.3	NA	NA
Asian or Pacific Islander	0.5	0.9	0.5
Black or African American	20.2	21.9	22.8
Hispanic or Latino	1.2	1.1	1.2
White	51.9	30.5	46.3
More than 1 race or ethnicity	0.2	1.3	0.5
Female			
American Indian and Alaska Native, non-Hispanic	NA	NA	NA
Asian or Pacific Islander	0.2	0.7	0.3
Black or African American	7.6	17.1	8.9
Hispanic or Latino	0.3	0.5	0.4
White	17.5	25.1	18.9
More than 1 race or ethnicity	NA	0.8	NA

Abbreviations: ASD, autism spectrum disorder; ID, intellectual disability; NA, not available.

^a^ Counts and some percentages are censored for compliance with cell suppression policy; all are less than 0.2%.

Youth with an ASD diagnosis had a 5% chance of being in foster care in the bivariate analyses (Table 3). After adjusting for age, the likelihood of a 0- to 5-year-old, White youth with ASD having foster care involvement decreased to 3%. Similar declines in foster care involvement, after adjusting for age, were observed across the groups with ASD and ID or ID only. The likelihood for foster care involvement increased with age in all groups relative to 0- to 5-year-old youth, although youth aged 13 to 17 years were more than twice as likely to have foster care involvement in all groups than the youngest age group. The odds of foster care involvement declined slightly after age 17 years, in the 18- to 21-year-old age group, among all diagnosis subgroups.

**Table 3.** Logistic Regression Models for Foster Care Involvement by Diagnostic Subgroup

Characteristic^a^	Adjusted OR (95% CI)								
	ASD only			ID only			ASD and ID		
Intercept	0.05 (0.05-0.05)	0.03 (0.03-0.04)	0.03 (0.03-0.03)	0.07 (0.07-0.07)	0.04 (0.04-0.05)	0.04 (0.03-0.04)	0.06 (0.06-0.06)	0.03 (0.02-0.04)	0.03 (0.02-0.03)
Age group, y									
6-12	NA	1.36 (1.28-1.44)	1.35 (1.27-1.43)	NA	1.59 (1.42-1.8)	1.59 (1.42-1.79)	NA	1.97 (1.54-2.55)	1.95 (1.53-2.53)
13-17	NA	2.06 (1.94-2.19)	2.07 (1.95-2.2)	NA	2.17 (1.93-2.44)	2.15 (1.92-2.42)	NA	2.57 (2.02-3.33)	2.56 (2.01-3.32)
18-21	NA	1.44 (1.34-1.55)	1.44 (1.33-1.55)	NA	1.27 (1.13-1.43)	1.26 (1.12-1.42)	NA	1.51 (1.18-1.97)	1.50 (1.17-1.95)
Sex									
Female	NA	NA	1.35 (1.29-1.4)	NA	NA	1.10 (1.05-1.15)	NA	NA	1.18 (1.1-1.27)
Race and ethnicity									
American Indian and Alaska Native, non-Hispanic	NA	NA	1.08 (0.82-1.38)	NA	NA	1.25 (0.78-1.88)	NA	NA	0.93 (0.46-1.67)
Asian or Pacific Islander	NA	NA	0.19 (0.16-0.23)	NA	NA	0.39 (0.32-0.46)	NA	NA	0.17 (0.12-0.24)
Black or African American	NA	NA	1.37 (1.32-1.42)	NA	NA	1.32 (1.26-1.39)	NA	NA	1.37 (1.28-1.47)
Hispanic or Latino	NA	NA	0.94 (0.81-1.07)	NA	NA	1.12 (0.93-1.34)	NA	NA	1.26 (0.96-1.61)
More than 1 race or ethnicity	NA	NA	0.77 (0.53-1.06)	NA	NA	1.35 (1.14-1.58)	NA	NA	1.06 (0.69-1.55)

Abbreviations: ASD, autism spectrum disorder; ID, intellectual disability; NA, not applicable; OR, odds ratio.

^a^ The reference value for age group was 0-5 y; for sex, male; and for race and ethnicity, White.

## Discussion

Population-level analyses quantifying foster care involvement of youth with I/DD found that in 2016 more than 39 000 youth with I/DD had foster care involvement, encompassing 4.3%, 4.6%, and 5.1% of the Medicaid population with ASD only, ASD and ID, or ID only. During 2016, a total of 437 465 youth resided in foster care,^27,28^ with our results using Medicaid claims indicating that nearly 8.9% of these youth had an intellectual or developmental disability. Our analysis of specific diagnosis groups found rates of ASD and ID among youth in foster care that were 2 to 5 times the rates found in the general population in the US. Collectively our findings indicate that youth with I/DD are overrepresented in the foster care system, and that among youth with I/DD, Black youth and females across all diagnosis groups were at an increased risk for foster care involvement, with the risk especially high among youth with an ASD-only diagnosis.

White males made up the majority of youth in each diagnostic subgroup (ASD only, ID only, and co-occurring ASD and ID), which is consistent with previous research on the general population.^18,23^ However, our findings that Black youth with I/DD face an increased risk of foster care placement align with research that has identified disparities and overrepresentation of Black youth at each point of contact with the foster care system, including placement in foster care, both in the United States and western European countries.^29,30^ The potential causes of the disproportionate involvement of Black youth in foster care have been grouped into 2 categories: differential treatment by child welfare agencies and other systems due to racial discrimination and differential experiences of Black families due to systemic and historical marginalization.^29,30,31^ Furthermore, the increased risk of foster care involvement among Black youth with I/DD may be attributed to the lack of policies and programs addressing the intersection of disability, race, and sex, along with the associated service and support needs within the foster care system.

The data from this study cannot identify specific causes for foster care involvement for youth with I/DD. However, our results identify future research and action steps for consideration. First, our findings point to the need for research on the reasons why youth with I/DD enter foster care and when the diagnosis is identified. Prior research has yielded mixed findings regarding the reasons youth with I/DD are placed in care, but all suggest that families of children with I/DD are not receiving the full continuum of resources needed to care for their child.^2,12^

Increased attention to what happens to youth with I/DD once they are placed in foster care is urgently needed. The iatrogenic effects of foster care placement may also be amplified for youth with I/DD because even small disruptions in health care can have significant negative effects on short-term and long-term health outcomes. This creates the possibility that foster care placement can exacerbate existing health disparities present in this population. Therefore, it is critical that health care professionals work with child welfare representatives and family members to maximize continuity of care for youth with I/DD placed in foster care. Health care professionals can also advocate for youth with I/DD to receive culturally responsive programs like Kinship Care and Family Group Decision Making, to optimize the stability of out-of-home placements and improve health outcomes while retaining their connections to their families and communities.

Increased risk for females has specific implications for the ASD group (both with and without ID) because of a growing call for research focused on the identification of ASD in females and acknowledgment of variation in their reported services needs and use.^32^ Females with ASD are diagnosed later compared with their male counterparts. This delay likely impacts the disparities found in this study as access to services often hinges on a diagnosis, which can be especially delayed for Black youth and females.^33^

Youth with ASD only were younger than youth with ASD and ID or ID only. It may be that youth with ASD who present without a cognitive challenge observed in ID are identified earlier because their symptoms may have behavior support implications and necessitate support to avoid emergency or crisis placements. It may also be that the development or growth of ASD-specific Medicaid programs, such as waivers, in states that do not require a formal ID diagnosis early in childhood present opportunities to gain access to additional services. However, only a few states offer such waivers and they often have enrollment caps, which can make accessing needed services without an accompanying ID diagnosis more challenging.

Findings from this analysis also revealed that a greater proportion of foster care–involved youth with co-occurring ASD and ID or ID only were transition-age youth (18-21 years). These diagnoses, alongside foster care involvement, may facilitate many long-term barriers to optimal outcomes in adulthood for this population. Without adequate support, adverse outcomes in adulthood may include an increased risk for health problems, increased premature mortality, psychiatric comorbidity, homelessness, unemployment, and substance use challenges.^34,35^ The service cliff experienced by transition-age youth with ASD is likely worsened by foster care involvement; therefore, there may be a need for new or amplified Medicaid-specific programs that support service navigation. As youth transition across settings, their service needs likely both persist and change, and continuity in health care professionals, supported transitions, and care planning is advantageous. Research on outcomes among this group is especially urgent as ASD prevalence has continued to increase, which has implications for children who were diagnosed in the early 2000s and are now entering early adulthood.

Among all youth with I/DD, the continued lack of representation in research underscores a parallel lack of information for the foster care system.^36^ Increased inclusion of Black, female, and older youth at a heightened risk for foster care involvement based on I/DD diagnoses can enhance research exploring structural vulnerabilities that disproportionately place these youth in care (eg, sociodemographic factors like age, socioeconomic status, geographic location, and living area). Including I/DD status in research on populations at heightened risk for foster care involvement would generate a more thorough picture of supports that facilitate prevention for placement among those most at risk for foster care involvement.

### Limitations

The typical limitations of claims-based research apply to this study. Care received outside of the Medicaid system was not observed. There is a misclassification risk for diagnoses in the Medicaid system, despite the use of validated algorithms. Previous studies have also pointed to an underidentification of youth with foster care involvement in Medicaid claims data,^37^ although updated validation estimates are not available. While this research provides a point-in-time snapshot of rates of I/DD, ASD, ID, and ASD and ID combined were examined by intersectional identities of race and ethnicity and sex, yielding small cell sizes that preclude a more compressive intersectional analysis of the likelihood of foster care and disability in the regression analyses. These sample size limitations disproportionately affect groups who may be at heightened risk for foster care involvement, such as American Indian, Asian, Pacific Islander, or Native American individuals. Future studies should prioritize adjustments for person-time across Medicaid enrollment periods, especially when examining the positive correlation between proportion of children in foster care and age.

### Implications

The use of an intersectional approach to generate a national profile of foster care involvement among youth with I/DD highlights the need for continued monitoring of the size and characteristics of these groups. Solutions to address the increased risk for Black children and females with I/DD in foster care should include the development of integrative data that link both child welfare and health data to ensure a more comprehensive assessment of risk and research on pathways into care that account for both disabilities and the socioeconomic features of families that play a critical role in foster care placement. Furthermore, examination of the specific risk factors faced by Black youth with I/DD in youth aged 13 to 17 years is critical. Findings underscore concerns about the disproportionate involvement of Black children in foster care driven by higher rates of poverty, racial bias and discrimination, child welfare system factors, and structural racism.^38^ Given the interconnectedness of these systemic and structural barriers, person-centered approaches are essential to understanding risk factors because multiple factors can operate in tandem, and identifying differential contributions is vital for developing health risk profiles for youth with I/DD in foster care.^39^ While the identification of I/DD has improved among Black children and youth generally, the increased risk of foster care involvement may signal gaps in the provision of resources for families to address related health and mental health challenges. Examinations of preventive services and resources for Black youth with I/DD can help address disproportionate entry into foster care and establish standards for equitable practices of service provision for this group.

## Conclusions

This study found that among youth with I/DD, Black youth and females faced higher risk for foster care involvement, and the likelihood of foster care involvement increased with age. Future research should shift toward elucidating risk factors for foster care placement among youth with I/DD, including co-occurring conditions, and the unique needs of this population, especially at the intersection of race, ethnicity, sex, and disability, are needed to advance health equity and ensure these groups are not left behind.

## Notes

Supplement. Data sharing statement
