# Supplementary material for: Foster Care Involvement Among Youth With Intellectual and Developmental Disabilities
Source: JAMA Pediatr. 2024 Feb 12;178(4):384–90. doi: 10.1001/jamapediatrics.2023.6580 (PMC10862267; doi:10.1001/jamapediatrics.2023.6580)
Supplement: Supplement. — Data sharing statement [file jamapediatr-e236580-s001.pdf]

Data Sharing Statement

Shea. Foster Care Involvement Among Youth With Intellectual and Developmental Disabilities. *JAMA Pediatr*. Published February 12, 2024. doi:10.1001/jamapediatrics.2023.6580

Data
Data available: No

Additional Information
Explanation for why data not available: National Medicaid claims data are governed by a data use agreement with CMS and distribution is not permitted.
